# Feasibility of therapeutic Chinese massage (tui na) for peripheral neuropathy among people with human immunodeficiency virus: findings of a pilot randomized controlled trial

**DOI:** 10.3389/fneur.2023.1148150

**Published:** 2023-12-01

**Authors:** Song Ge, Linda Dune, Minhui Liu, Guojing Fu, Haixia Ma, Jiale Hu, Xuechun Lin, Junxin Li

**Affiliations:** ^1^Department of Natural Sciences, University of Houston-Downtown, Houston, TX, United States; ^2^Xiangya School of Nursing, Central South University, Changsha, China; ^3^Department of Tuina, Shandong Provincial Hospital Affiliated to Shandong First Medical University, Jinan, Shandong, China; ^4^School of Nursing and Health Studies, Hong Kong Metropolitan University, Hong Kong, China; ^5^Department of Nurse Anesthesia, College of Health Professions, Virginia Commonwealth University, Richmond, VA, United States; ^6^Hubei Key Laboratory of Food Nutrition and Safety, Department of Nutrition and Food Hygiene, School of Public Health, Tongji Medical College, Huazhong University of Science and Technology, Wuhan, Hubei, China; ^7^Johns Hopkins University School of Nursing, Baltimore, MD, United States

**Keywords:** HIV, peripheral neuropathy, massage, pain, randomized controlled trial, physical functioning, lower extremity functioning

## Abstract

**Background:**

Peripheral neuropathy (PN) is prevalent in people with human immunodeficiency virus (PHIV) with no Food and Drug Administration-approved treatment. Therapeutic Chinese massage (TCM) is a promising noninvasive and non-harmful intervention for HIV-related PN. However, relevant research is lacking. The purpose of this study is to evaluate the feasibility of TCM for HIV-related PN.

**Method:**

We conducted a pilot, single-centered, two-arm, double-blinded, randomized controlled trial. Twenty eligible PHIV were recruited primarily from the AIDS Foundation Houston, Inc. in Texas and were randomly assigned into two groups. Ten participants in the intervention group received three weekly 25-min TCM sessions by a certified TCM therapist. The remaining ten control group participants received the same therapist’s three weekly 25-min placebo massage sessions on their lower extremities. The outcome was the feasibility of this study as measured by recruitment and completion rates, participant safety, and treatment adherence and compliance, as well as the effect size of the intervention.

**Results:**

The study population comprised 20 PHIV (mean age 55.23). This study showed high feasibility as measured by a high rate of recruitment, a 100% rate of completion, and zero serious adverse events. As we inquired 21 respondents for eligibility for the study, all except one had HIV-related PN. All respondents were willing to participate in the study and adhered to the group assignment after they enrolled in the study. The participants’ baseline pain was at a medium to a high level (6.30 [2.15] out of 10).

**Conclusion:**

Chinese massage is a feasible intervention in PHIV. Future relevant randomized controlled trials are expected.

**Clinical trial registration:**

https://clinicaltrials.gov/, NCT05379140.

## Introduction

Human immunodeficiency virus (HIV), a retrovirus that leads to a person’s progressive immune system failure, affects about 1.2 million people in the US (1). People with human immunodeficiency virus (PHIV) have had significantly improved survival owing to the advancement of combination antiretroviral therapy (ART) (2). Specifically, between 2002 and 2007, PHIV on ART in the US have increased their life expectancy by 15.3 years. In 2013, 20-year-old PHIV on ART in the US were expected to live into their early 70s, a life expectancy approaching that of the general population (3, 4). Thus, HIV has gradually become a chronic condition requiring lifelong therapy and symptom management in the US (5).

As the most common neurological manifestation in PHIV, peripheral neuropathy (PN) is related to irreversible damage to sensory nerves and affects 30–67% of PHIV (6, 7). HIV-related PN can be caused by the HIV or neurotoxic effects of early ART, especially nucleoside reverse transcriptase inhibitors (8). Patients who were exposed to the early regimens may have residual effects. The symptoms of PN include numbness, loss of sensation (often in the toes and soles), paresthesia, burning sensation, and stabbing pain in the extremities (9). HIV-related PN most often affects the feet and, less often, the hands (8). PN negatively affects the quality of life in PHIV and is associated with an increased risk of depression, anxiety, insomnia, and high plasma triglyceride in PHIV (10, 11). Currently, there is no US Food and Drug Administration-approved treatment for HIV-related PN. PHIV often take off-label-use analgesics, anti-depressants, or anti-seizure drugs for symptom reduction. These medications only temporarily alleviate PN symptoms to a limited extent and may cause nausea, vomiting, dizziness, and fatigue that disturb PHIV (12). Evidence-based pharmacological interventions are rare. A systematic review and meta-analysis of randomized controlled trials on pharmacological interventions to treat HIV-associated sensory neuropathy pain only found marginally significant efficacy in smoked cannabis, topical capsaicin, and recombinant human nerve growth factor (8).

Research studies have supported pharmacological and complementary approaches to addressing PN in PHIV (13). However, very few studies examined nonpharmacological interventions to treat HIV-related PN. To the best of our knowledge, previous randomized control trial interventions on non-pharmacology interventions to treat HIV-related PN exclusively included hypnosis (14), spinal cord stimulation (15), lower extremity splinting (16), a vibratory stimulus (17), progressive-resisted exercises (18), cognitive behavior therapy (19), and Acupuncture/Moxibustion (20). Of the studies above, only the Acupuncture/Moxibustion study showed marginally significant pain reduction over the placebo.

Therapeutic Chinese massage (tui na) (TCM) has over 5,000 years of practice history in China (21, 22). It includes systematic manual palpation, push-pull, and stroking of soft tissues and muscles delivered by a therapist’s fingers, hands, elbow, knee, or feet. It also includes manipulation of the acupoints (23). The resulting pressure on the muscles and joints of the foot and lower leg may promote blood circulation and stimulate the somatosensory system (24). TCM has been shown to reduce chronic low back pain and neck pain (25, 26) and if combined with hypertensive drugs, helps better alleviate hypertension than hypertensive drugs alone in adults (27). In addition, massage is mostly non-harmful if delivered by a qualified therapist and is noninvasive.

Limited research studies with small sample sizes on the effects of massage on PN have focused on diabetes-related PN and showed the beneficial effects of massage (24, 28). To our knowledge, there is no study evaluating the effectiveness of TCM on HIV-related PN. Thus, the purpose of this pilot randomized controlled trial was to evaluate the feasibility of this design by measuring recruitment and completion rates, participant safety, and treatment adherence and compliance.

## Method

### Ethical approval

The University of Houston-Downtown Committee for the Protection of Human Subjects ethically reviewed and approved this study with approval No. CPHS #44–21. The AIDS Foundation Houston, Inc. provided non-financial-related support to this study. Written informed consent was obtained from the participants before they enrolled in the study. The informed consent contained information on voluntary participation, withdrawal, study risks, confidentiality, and secured data storage. This trial was registered with ClinicalTrials.gov of the National Institute of Health in May 2022 (ClinicalTrials.gov Identifier: NCT05379140).

### Study design

This study is a pilot, single-center, two-arm, double-blinded randomized controlled trial. Twenty participants were randomly assigned to either the TCM group or the placebo massage group in a one-to-one ratio. Ten participants in the TCM group received three weekly 25-min TCM sessions. The rest ten participants in the placebo massage group received three weekly 25-min placebo massage sessions. The study took place at the participants’ homes or in a room provided by the AIDS Foundation Houston, Inc. based on the participants’ convenience and preferences. The AIDS Foundation Houston, Inc. provides permanent, safe, and affordable living and other supportive services to PHIV. The study followed the Consolidated Standards of Reporting Trials (CONSORT) Statement (29). Due to this being a pilot study, we used a convenient sample from HIV Foundation Houston, Inc.

### Inclusion criteria and recruitment

The study inclusion criteria were people who (1) had an HIV diagnosis confirmed by the HIV Foundation Houston, Inc.; (2) self-reported PN-related symptoms in their lower extremity, including sharp, jabbing, throbbing or burning pain, numbness, decreased sensation to pinprick, prickling or tingling feeling, lack of coordination and falls, muscle weakness, and extreme sensitivity to touch (30); (3) were not taking any medications to alleviate PN; (4) aged at least 18 years old; (5) spoke English or Mandarin Chinese; (6) could give informed consent; (7) were not pregnant or lactating; (8) were not enrolled in other research studies. People who had received any kind of massage in the past 7 months were excluded from participation.

This research study was publicized using flyers, officers’ referrals, and the snowball referral method of enrolled participants. Individuals interested in this study were encouraged to contact the principal investigator via email or phone for an eligibility check. The principal investigator conducted an initial screening of each interested respondent over the phone. She asked them whether they were having PN symptoms in their lower extremity, including sharp, jabbing, throbbing, or burning pain, numbness, decreased sensation to pinprick, prickling or tingling feeling, lack of coordination and falling muscle weakness, and extreme sensitivity to touch. If they did have PN symptoms, the principal investigator would schedule a time to meet with them individually at their home or a room provided by the HIV Foundation Houston, Inc. at their convenience and preference. When the principal investigator met them in person, she conducted a full screen based on the inclusion and exclusion criteria, determined their suitability for participation and invited the eligible PHIV. Following informed consent, 20 PHIV were recruited between December 2021 and May 2022. After completing the study, each participant received a visa gift card of $70 for their time.

### Randomization

In this study, the participants were randomly assigned to either the TCM or the placebo massage group in a one-to-one ratio using a randomized blocked design (block size 4) using R statistical software (version 2020) (31). This effort ensured that each group had a similar number of participants throughout the recruitment process until the desired sample size was reached. After the participant consented to enroll in the study and the baseline data were collected, the therapist was notified of the group assignment. Information of participants in each group was deidentified. A unique identifying number was used for further analysis. All information was kept confidential. Only authorized persons had access to it.

### Blinding

This is a double-blinded study. While the principal investigator and the therapist knew the participants’ allocation, the participants, the statistician, and the outcome assessors were blinded.

### The TCM (tui na)

Ten participants in the TCM group received three weekly 25-min TCM sessions. Each session included an assessment of the legs and toes of the affected extremity for broken skin and lesions. The therapist would try to avoid these areas. The participant would be positioned with support to the foot and legs with the sole of the foot directed downward and the provider directly in alignment with the soles of the foot. The therapist sequentially performed the following four steps for each TCM session. Table 1 described the TCM points of the TCM group.

**Table 1:** The TCM points of the TCM group.

Channel	Acupoints	Location	Technique	Manipulation times	Purpose of point
Gall Bladder (GB)	GB 34	Depression anterior and inferior to the fibular head	One finger-pressing with thumb tip flexion and extension and fingertip-pushing	Three treatments of 15 s each	Promotes flow of Liver Qi and relaxes sinews and muscles
	GB 35	7 cun from popliteal crease, posterior border of the fibula	As above	As above	Relaxes sinews, muscles, and stops pain
	GB 36	7 cun from popliteal crease, anterior border of the fibula	As above	As above	Removes channel obstruction and stops pain
	GB 37	Down 2 cun from GB36	As above	As above	Relieves motor impairment of and pain in lower extremity
	GB 38	Down 1 cun from GB37	As above	As above	Relieves pain in lower extremity
	GB 39	Down 4 cun from GB 35, posterior border of the fibula	As above	As above	Relieves spastic pain in lower leg
	GB 40	Inferior and anterior to lateral malleolus, depression lateral side of tendon extensor digitorum longus.	As above	As above	Relieves pain in lower leg, ankle, and foot
	GB 41	Junction of fourth and fifth metatarsal bones on lateral side of tendon extensor digitorum longus.	Major thenar kneading manipulation	Following GB 34 through 40 manipulation for 30 s	Relieves spastic pain in lower leg and foot
Kidney (KI)	KI 1	The sole of the foot, one third distance from base of second toe to the posterior edge of the heel.	Rolling the bottom of the foot to include pressing manipulation with interphalangeal joint of middle finger to K 1	15 s for three rolling and pressing manipulations.	Restores microcirculation and treat nerve disorders
Spleen (SP)	SP 5	Depression distal and inferior to medial malleolus	Rubbing	Five seconds or until area warm to touch	Relieves pain in foot and ankle

In order to encourage restoration of neuron function and decrease pain, acupressure techniques seek to enhance ion channel activity, decrease oxidative stress, neuron inflammation and restore mitochondrial functioning (32, 33).

Step one: To begin, using the thumb tip of the dominant hand, the therapist applied pressure and deep up and down movements to create vibration directly to the acupoints along the lower extremity Gall Bladder Channel (GB) points; Qiuxu (GB40), Juegu (GB39), Yangfu (GB38), Guangming (GB37), Waigu (GB36), Yangjiao (GB 35), and Yanglingquan (GB34) (Table 2). All pressing and vibration maneuvers were repeated for three rotations of 60 s per point with the same sequence along the channel. The vibrating massage delivered 50 strokes over 30 s per point.

**Table 2:** Participants’ baseline characteristics.

Characteristics	Total (*n* = 20)	TCM group (*n* = 10)	The placebo group (*n* = 10)	*p* value
Age (mean, SD)	55.23 (8.29)	50.91 (8.93)	60.61 (5.16)	**0.010**
Male (*n*, %)	9 (45.0)	5 (50.0)	4 (40.0)	>0.999
Living alone (*n*, %)	20 (100.0)	10 (100.0)	10 (100.0)	1.000
Unemployed (*n*, %)	18 (90.0)	9 (90.0)	9 (90.0)	1.000
Black (*n*, %)	19 (95.0)	9 (90.0)	10 (100.0)	1.000
White	1 (5)	1(10)	0 (0)	
Others	358 (4.1)	316 (4.4)	42 (3.0)	
0–12 years of education (*n*, %)	10 (50)	4 (40.0)	6 (60.0)	0.714
12–16 years of education	7 (35.0)	4 (40.0)	3 (30.0)	
16 and above years	3 (15.0)	2 (20.0)	1 (10.0)	
Neuropathy pain rate, mean (SD)	6.30 (2.15)	6.00 (2.31)	6.60 (2.07)	0.548
The Lower Extremity functioning score, mean (SD)	53.47 (18.28)	58.11 (24.43)	49.30 (9.87)	0.335
Physical functioning score, mean (SD)	52.50 (28.68)	41.00 (29.98)	64.00 (23.31)	0.071
Bodily pain score, mean (SD)	52.45 (29.73)	50.20 (34.24)	54.70 (26.12)	0.745
General health score, mean (SD)	61.40 (21. 44)	62.80 (23.49)	60.00 (20.36)	0.779

SD means standard deviation. *N* (%) means number and percentage.

Bolded values mean statistical significance (*p* < 0.05).

Step two: The therapist and participant identified the point experienced as most painful during the pressure and vibration technique under step one. The sensitive areas included painful points, contracted muscle areas, and nodules. The therapist used the thumb of the dominant hand to elicit subjective responses of either numbness, decrease in pain, or relief of pain while applying deeper penetrating pressure at the point identified.

Step three: The technique of rolling was used to the plantar aspect of the affected foot. The technique of rolling began with a clinched fist that progressed through a rolling-type movement with fixed hand placement commencing with the palm facing upward. This technique is a sequence of contact between the third though fifth fingers of the therapist’s dominant hand to the plantar aspect of the foot for three passes from the heel to the toes. Step three concluded with pressing and vibrating manipulation with the interphalangeal joint of the middle finger of the therapist to the participant’s Yongquan (KI 1) acupoint.

Step four: The kneading manipulation used soft and penetrating movements to encourage local heat generation. This technique of three repetitions was performed with the palm of the therapist’s hand applying pressure to the underlying muscles at GB 40, Zulinqi (GB 41), and the plantar aspect of the foot. Further treatment to the foot included shaking of the ankle by grasping the sides of the ankle with both hands and gently moving the foot up and down for 10 movements and rotating the ankle to full range of motion as tolerated by the participant. Finally, toe rotation and gentle pulling of each toe were followed by rubbing of Shangqiu (SP 5) until the area was assessed by the therapist as warm to touch and the participant reported a warm sensation over the medial malleolus.

### The placebo massage

Ten participants in the placebo massage group received three weekly 25-min placebo massage sessions that included (1) an assessment of the legs and toes of the affected extremity for broken skin and lesions for the purpose of avoiding them during the massage, (2) gentle light touch massage to the foot and toes without point stimulation or other techniques of TCM. The rest ten participants in the control group were offered the opportunity to receive the TCM intervention after this study was completed.

### The TCM/placebo massage therapist

The therapist in this study is a certified Chinese massage therapist and a registered nurse with over 10 years of clinical experience as a Diplomate in Asian Body Works (NCCAOM). The therapist provided the TCM and the placebo massage for both groups. Prior to beginning this study, she completed a three-hour training to master the research protocol with the principal investigator and passed an examination where she recited the protocol verbally and demonstrated each TCM technique to the principal investigator.

### Safety

During the TCM or placebo massage, the therapist started by assessing the legs and toes of the affected extremities of the participants for broken skin and lesions and tried to avoid them during the massage. Moreover, the therapist asked participants if they felt increased discomfort such as pain, increased sensitivity of the feet, tenderness, nausea, sweating, rashes on the skin, and extreme tiredness during the massage. If the participants reported any increased discomfort or pain, the therapist would provide the massage more gently. As the treatment sessions proceeded, the therapist evaluated any unfavorable responses from the participants and followed them until the problem was resolved. These events would be carefully recorded in the case report form by the therapist. The therapist wore latex or nonlatex (for people allergic to latex) gloves when delivering TCM/placebo massage and wash hands after each session.

### Measures

The principal investigator trained two outcome assessors on the study data collection protocol prior to any data collection.

## Sociodemographic information

Participants’ sociodemographic information was collected through a standardized self-report questionnaire via an iPad on their first visit. The questionnaire included age (years), race (self-identified as African American or not), gender (male or female), education (0–6, 6–12, 12–16, and above 16 years of education completed), partner status (living with a partner or not), and employment status (employed or not).

The outcome of this study was the feasibility of TCM on PHIV with PN. Feasibility measures included rate of recruitment and completion (No. of referred, No. of eligible, No. of enrolled, No. of withdrawals, study recruitment rate, and study completion rate), patient safety (No. and severity of adverse events), treatment adherence (range and average time of message session, No. of completed sessions and missed sessions) and compliance (No. of completing the study in the originally assigned group). The therapist was instructed to pay particular attention to the adverse events as reported by a systematic review of adverse events of massage therapy in pain-related conditions, including soreness, soft tissue trauma, neurologic compromise, bone fracture, hematoma or hemorrhagic cyst, syncope, cauda equina syndrome, pain, and dislocation (34).

In addition, to calculate effect size, PN-related pain, lower extremity functioning, and health-related quality of life were measured prior to (at the first visit, before the first TCM/placebo massage session) and after the intervention (at the third visit, after the third TCM/placebo massage session) for all the 20 participants.

PN-related pain was measured by the numerical pain rating scale (35). A score of zero indicated “no pain at all” and a score of ten meant “the worst pain ever possible.” The scale had good reliability and validity in assessing chronic pain (36).

Lower extremity functioning was measured by the Lower Extremity Functional Scale (LEFS) (37). The LEFS not only had good internal reliability, test–retest reliability, and cross-sectional construct validity (38), but also was able to detect accurate and clinically meaningful changes among individuals with lower extremity musculoskeletal conditions (39).

Health-related quality of life was measured by the Medical Outcomes Study Questionnaire Short Form 36 Health Survey (SF-36) (40). A lower score in the SF-36 indicated more disability and a higher score indicated less disability. The SF-36 contained eight subscales on various domains of quality of life. Each subscale had a score ranging from 0 to 100. For the purpose of this study, we only focused on SF-36 subscales on physical functioning, bodily pain, and general health. For physical functioning and general health scores, a higher score indicated better physical functioning or better general health, respectively. For bodily pain, a higher score indicated worse bodily pain. The reliability of the SF-36 was >0.80 (40, 41).

### Data analysis

The characteristics of the 20 participants were summarized by treatment group. We calculated means (standard deviation) for normally distributed continuous data and medians (interquartile range) for non-normally distributed continuous data. We calculated the frequency (percentages) for categorical data. T-tests and Fisher’s exact tests were used to examine group differences for continuous variable and categorical variables, respectively.

For the outcome on feasibility, rates of recruitment (a proportion, defined as the number of consented individuals/numbers of eligible individuals), rate of completion (undertaken baseline and follow-up tests), adherence (participants’ completed sessions/number of sessions), and adverse events (total number and number per participant) were calculated. The effect sizes were calculated using methods proposed by Cohen (42). Based on this method, if the two groups had the same sample size, the effect size was calculated by subtracting the means and dividing the result by the pooled standard deviation. The resulting effect size, Cohen’s *d*, was the difference between the two groups in terms of their common standard deviation (43).

The statistician was independent of the research team but was blinded to the group assignment. All analyses were performed using R statistical software (version 2020) (31). A two-sided value of *p* of 0.05 or less was considered significant.

## Results

The baseline characteristics of the 20 participants were described in Table 2. Most of the participants were female (55%), African Americans (95%), living alone (100%), and not employed (90%). The participants’ mean age was 55.23 years (SD = 8.29). Sixty percent of the participants completed six to twelve years of education. At baseline, the mean numerical pain rating of the 20 participants was 6.30 out of 10, indicating moderately strong pain. Their mean LEFS score was 53.47. There was no statistical difference in the baseline characteristics between the two groups except for age. The participants in the control group were significantly older than those in the experimental group (mean age = 60.61 and 50.91, respectively; value of *p* = 0.01).

A total of 21 respondents contacted us and showed interest in our study. Eighteen of them lived in homes provided by the AIDs Foundation Houston, Inc. Two were referrals of people living in homes provided by the AIDs Foundation Houston, Inc. One respondent had no PN symptoms and thus was excluded from participation. Two participants used to take pain medications for managing PN-related pain but were not taking pain medications at the time we recruited them. Thus, we determined that they were eligible for participation—a total of 20 participants enrolled in the study. No one quit the study or did not adhere to the group assignment. The participants did not report any severe adverse events other than soreness in the massage area. All 20 participants completed the TCM/placebo massage as assigned and as scheduled with no missed sessions (Table 3).

**Table 3:** Differences in the score change from baseline to post-intervention between the TCM and the placebo group for different outcomes.

Outcome		Difference	95% CI	P value
Numeric pain rating	Model 0	−0.4	−3.38, 2.58	0.79
	Model 1	−0.84	−3.82, 2.13	0.57
LEFS score SF-36 Physical functioning SF-36 Bodily pain SF-36 General Health	Model 0 Model 1 Model 0 Model 1 Model 0 Model 1 Model 0 Model 1	−18.27 −14.01 19.50 23.15 −6.9 −7.19 0.9 −1.95	−47.9, 11.37 −42.42, 14.39 −3.35, 42.35 0.19, 46.11 −35.12, 21.32 −35.89, 21.51 −17.18, 18.98 −20.98, 17.08	0.22 0.32 0.09 0.05 0.62 0.61 0.92 0.84

Model 0 was unadjusted. Model 1 was adjusted for age.

Using Cohen’s method, the effect size Cohen’s *d* (95% confidence interval) for the numerical pain rating, LEFS score, SF-36 physical functioning subscale, SF-36 bodily pain subscale, and SF 36 general health subscale was −0.335 (−1.218, 0.547), −0.856 (−1.772, 0.060), 0.335 (−0.548, 1.218), −0.043 (−0.919, 0.834), and −0.144 (−1.021, 0.734), respectively. All the five Cohen’s *d*, except the one on LEFS score, were less than 0.2 and considered a small effect size (42).

## Discussion

The participants of this pilot randomized controlled trial were predominantly middle-aged and unemployed African Americans who lived alone. We found the high feasibility of the study design. Powered randomized control trials should be conducted in the future to overcome the limitations of this study and fill the research gap.

In terms of feasibility, this study showed high feasibility as measured by a high rate of recruitment, 100% rate of completion, and zero serious adverse events. As we inquired 21 respondents for eligibility for the study, all except one had HIV-related PN. All respondents were willing to participate in the study and adhered to the group assignment after they enrolled in the study. One possible explanation is that most participants (18 out of 20) were living in homes provided by the AIDS Foundation, Houston, Inc. They were at an advanced stage of HIV to qualify for the housing program and thus had a higher prevalence of PN than general PHIV. The participants’ baseline pain was at a medium to high level (6.30 [2.15] out of 10). Many participants had problems with activities of daily living such as climbing stairs, going to work, or doing housework as evidenced by an LEFS score of 53.47 (18.28) out of 100 and an SF-36 physical functioning score of 52.50 (28.68) at baseline. Thus, they were looking for nonpharmacological interventions to manage their pain and other PN symptoms and restore their life functioning. When they received the TCM, they spoke highly of it. Most of the participants in the TCM group mentioned that the massage made them feel more comfortable. In addition, the participants lived in two housing areas, knew each other well, and supported each other to enroll and complete this study. In terms of safety, TCM was shown to be safe for PHIV. To start with, we hired a highly experienced nurse and therapist in TCM. Besides doing the TCM/placebo massage, she kept a careful recording of any adverse events that happened. The only complaint recorded was soreness and pain in the massage area, but the level was tolerable. This is a common reaction for people receiving TCM. The symptoms were quickly relieved. Thus, the study design is highly feasible and TCM is safe and desirable for PHIV.

The major limitation of this study is that we recruited participants from one institution only; thus, the participants knew each other, which led to possible unblinding and potential group dynamics (e.g., discussion of intervention received and supporting each other). Another limitation of this study is underpowered with a small sample size. These limitations could bias our results and limit the generalizability of our results. In addition, we did not incorporate measurement tools that are tailored to HIV-related PN, such as the brief peripheral neuropathy screen (44), the subjective peripheral neuropathy screen (Subjective-PNS) (45), and the single question neuropathy screen, and quantitative sensory testing (QST) (46). Last, we did not assess long-term PN-related outcomes after the TCM/placebo massage due to time constraints. Future studies can utilize more tailored measures, measure the long-term effects of TCM/placebo massage, and include a larger sample size based on the effect sizes generated from this study.

There are several strengths of this study. To start with, this study focuses on an important but understudied area of research. With PHIV living longer (47), safe and effective treatments to improve their quality of life-related to prevalent conditions are likely to be increasingly important. However, a systematic review of pharmacologic and non-pharmacologic interventions for HIV-neuropathy pain only found seven non-pharmacological interventions for HIV-neuropathy pain with a total of 742 participants (48). Among them, only the Acupuncture/Moxibustion study showed marginally significant pain reduction in the experimental group over placebo. Thus, although HIV-related PN is prevalent in PHIV, it is barely studied and overlooked in research. In addition, we calculate the effect size of the TCM on various outcomes of PHIV and provide implications for powered randomized controlled trials.

To our knowledge, no study has examined the effectiveness of TCM on HIV-related PN. This pilot study raised awareness of the research gap and provided in-depth insights into the design, feasibility, and implications of such a study design by providing a clearly written and detailed pilot study procedure and results. Moreover, the TCM we propose is relatively safe. In a systematic review of adverse events of massage therapy in pain-related conditions, researchers found that serious adverse events associated with massage in general and pain-related massage are very few (34). Another strength of our study is our study population who is predominantly middle-aged unemployed African Americans who were living alone. They are a marginalized group in society and are disproportionately affected by HIV with higher mortality than other ethnic groups (49). Thus, our study targets a marginalized population. Finally, we calculated effect sizes for several outcomes which serve as a foundation for future larger randomized controlled trials on this topic.

It was worth noting that after the collection of the outcomes, the therapist provided teaching to encourage and support lifestyle changes, such as methods of performing foot massage at home and the importance of keeping feet warm all the time for the 20 participants. The therapist instructed the participants to perform toe flexion exercises, kneading massage to the foot including the locations of GB 40 and 41, and rolling manipulation to the sole of the foot with a provided racquetball for pressure to KI 1. The research team planned to follow up with the participants for a future study to determine the long-term relief of the neuropathy symptoms.

## Conclusion

This study showed that Chinese massage is a feasible intervention in PHIV. Future relevant randomized controlled trials are expected.

## Data availability statement

The raw data supporting the conclusions of this article will be made available by the authors, without undue reservation.

## Ethics statement

The studies involving humans were approved by University of Houston IRB committee. The studies were conducted in accordance with the local legislation and institutional requirements. The participants provided their written informed consent to participate in this study.

## Author contributions

SG, GF, LD conducted the study and drafted the study. XL conducted data analysis. JL, HJ, MH, and LM revised the manuscript. All authors contributed to the article and approved the submitted version.
